# Metabolomics in Adult and Pediatric Nephrology

**DOI:** 10.3390/molecules18054844

**Published:** 2013-04-24

**Authors:** Vassilios Fanos, Claudia Fanni, Giovanni Ottonello, Antonio Noto, Angelica Dessì, Michele Mussap

**Affiliations:** 1 Neonatal Intensive Care Unit, Puericulture Institute and Neonatal Section, Azienda Ospedaliera Universitaria, Cagliari 09131, Italy; 2 Department of Surgical Sciences, University of Cagliari, Cagliari 09131, Italy; 3 Department of Laboratory Medicine, IRCCS AOU San Martino-IST, University-Hospital Genoa, Genoa 16132, Italy

**Keywords:** metabolomics, kidney, adult, children, nephrology

## Abstract

Metabolomics, the latest of the “omics” sciences, has a non-selective approach and can thus lead to the identification of all the metabolites (molecules < 1 kDa) in a biological system. The metabolomic profile can be considered the most predictive phenotype capable of evaluating epigenetic modifications determined by external factors. It is so close to the phenotype as to be considered the phenotype itself in its unique individuality (fingerprinting), both in health (phenome), and disease (diseasome). Urine, compared to other biological liquids, has the advantage of being a complex fluid with many components, including intermediate metabolites. Metabolomics may thus play a role in the study of different kidney diseases and overcome diagnostic difficulties. We shall present the studies that to our knowledge have been published on Nephrology and Pediatric Nephrology. Some are experimental while others are clinical. We have not considered carcinomas and transplantations. Although scarce, the data on adults and the very few ones in pediatrics are quite interesting. Further studies on kidneys are needed to determine the practical clinical impact of metabolomics in kidney renal pathologies. The “multiplatform” “omic” study of urine and namely metabolomics can contribute to improving early diagnosis and the outcome of kidney diseases.

## 1. Introduction

The advent of the “omics” sciences (genomics, transcriptomics, proteomics, metabolomics) has revolutionized the approach to the study of biology and pathology. Today, the trend is towards “multiplatform” studies corresponding to one or all of these holistic approaches, which are both extremely innovative and promising.

The genetic code can be understood as a hereditary predisposition to respond in a given way to a disease, a drug or environmental changes, or develop a given pathology (what may occur). It does not, however, allow evaluation of the changes in the organism as the result of factors outside it, for example diet, drugs or the environment.

The study of the genetic expression is transcriptomics, which describes the whole of the mRNA present in a cell or tissue over time. Proteomics is the study of the translation of the proteins, while the proteome is the complete set of proteins expressed in a cell or tissue at every single instant. Integration of the information provided by these sciences contributes substantially to our understanding of the molecular processes involved in the physiological mechanisms in the state of health and their destruction connected with a disease. Expression of the protein and the mRNA can be evaluated only after hours, days or weeks from the time of their production.

Metabolomics is the latest of the “omics” sciences, defined in different ways [1,2,3], but one among the most quoted definitions is that by Nicholson as the quantitative analysis of all metabolites to be found in a biological sample [1]. The novelty of metabolomics emerges from the number of works published on this subject: in the last four years some ten thousand publications have appeared in PubMed, having as their subject metabolomics, metabonomics and metabolic profiling.

## 2. Metabolomics and Systems Biology

Metabolomics has a non-selective approach and can thus lead to the identification of all the metabolites (molecules < 1 kDa) in a biological system [2]. Thus, metabolites are not a static final product, but dynamic biocomponents that interact continuously with one another [3]. Metabolites, as intermediaries in biochemical reactions, can be seen as the final product of the genic expression or proteic and enzymatic activity; their study allows us to trace the metabolic profile of a biological system [4,5]. Since most of the urine metabolites are waste products, it is like “rummaging about in the waste products”. It is possible to find a great deal of information about who produced such substrates However, it must be underlined that the metabolome is not composed of waste products: in fact most of the metabolites in the body are necessary substrates for active metabolic pathways.

Unlike the other “omics” sciences, such as genomics and proteomics, metabolomics is a dynamic study that reflects the phenotype of an organism at a specific time. Metabolomics completes the information provided by the first investigative levels: since metabolism is regulated by signaling molecules and enzymes, the gene expression adapts first, then it changes the metabolic processes and finally the concentrations of metabolites. Such modifications are context-dependent, varying on the basis of the development and the physiological or pathological state of a cell, a tissue or an organism. Thus the metabolic profile can be considered the most predictive phenotype capable of evaluating epigenetic modifications determined by external factors [6].

The metabolic expression is the result of the interaction between the several “multiplatform” biological processes, since a single metabolite may derive from different metabolic processes interconnected at different levels and with numerous mechanisms of reciprocal regulation. Its evaluation allows the study of such processes not as isolated phenomena but as part of a network, thus leading to an overall understanding of the biological system [7]. By means of metabolic analyses we have thus gone from a reductionist vision to a holistic approach (Systems Biology) to the organism and an integrated understanding of its state of health or disease. Systems biology can in fact be defined as the quantitative analysis of the dynamic interaction of different components of a biological system through the combination of mathematical models of experimental biology. It considers the interaction of DNA-RNA-proteins and metabolites, and studies the network of relationships in which they are involved. This network characterizes the information flow at every level of biomolecular organization [3].

At present, approximately 2,900 endogenous metabolites can be identified, but if we consider their total number, estimated at 6,500 molecules, this is lower than the 30,000 genes, the 50,000 mRNAs and the more than 100,000 proteins present in the human organism [8,9].

They are easier to study than the vaster “systems” (about 3,000 metabolites instead of 30,000 genes and 100,000 proteins). This lays the groundwork for simplification in the analysis of the information gained.

Metabolomics also allows comparison of the metabolic profiles acquired in the same individual or groups of individuals at different times. 

Briefly stated, metabolomics is a quick indicator of environmental modifications (seconds instead of hours and days); If we do not consider the cost of the initial equipment, it is economical given the possibility of analyzing hundreds of samples per week with a much lower cost per sample and also closer to the phenotype in that it also takes epigenetics into consideration: it is so close to the phenotype as to be considered the phenotype itself in its unique individuality (fingerprinting), both in health (phenome), and disease (diseasome).

The metabolic profiles identified by means of different analytical platforms (including mass spectrometry or nuclear magnetic resonance, which are the most popular methods), and analyzed through multivariate analysis of the results. However, any study that measures a comprehensive metabolic profile can be considered metabolomics, regardless of the analytical platforms. 

Metabolomics can use any biological fluid (blood, liquor, saliva, sweat, sperm, amniotic liquid, urine and so on) as a sample. Urine, compared to other biological liquids, has the advantage of being a complex fluid with numerous components, including the intermediate metabolites, but its collection in the case of neonates, especially extremely low birth weights (ELBW), may be particularly difficult. For this reason, urine samples are usually taken with a cotton ball, from which the urine is extracted by means of a syringe. The sample is then immediately conserved at −80 °C. The advantages of this way of collection samples are evident: it is noninvasive, simple, economical and appears to be reliable. 

## 3. Metabolomics in Nephrology

Kidney disease is one of the most significant emerging chronic conditions in the world, and as such is becoming the center of therapeutic development alongside diabetes and cardiovascular disease.

The basic sciences have made an important contribution to the analysis of the molecular mechanisms involved in kidney diseases, but the transposition of such discoveries into clinical practice remains difficult [10].

Kidney diseases are difficult to diagnose at an early stage, to be followed in progression and in evaluating response to therapy; all this increases the complexity and risk of a clinical trial [11]. Moreover, biopsies are greatly limited by their invasiveness and this limit is accentuated in pediatrics [12]. Owing to such limitations, we must find more sensitive, reliable and preferably noninvasive markers of kidney function [13]. The classic markers, such as the blood urea (azotemia) and serum creatinine, lack sensitivity and reveal kidney damage when an important nephronic loss has already taken place [14]. The best biomarkers are accurate, sensitive, relatively noninvasive and easy to use both at the patient's bedside and at the outpatient level [11].

Given urine’s complexity, with many components including intermediate metabolites, and therefore its suitability for analysis, metabolomics may play a role in the study of different kidney diseases and overcome diagnostic difficulties. For instance, NMR-based metabolomics is capable of following the metabolism in different areas of the kidney and thus obtain important information concerning nephrotoxicity [15]. We shall present the studies that to our knowledge have been published on the subject. Some are experimental while others are clinical. We have not considered carcinomas and transplantions.

### 3.1. Experimental Studies

The study by Sieber *et al.* presents a comparative analysis of new, noninvasive kidney biomarkers and metabolomic changes in male murine models of nephrotoxicity induced by gentamicin.

In the cortex of the treated animals an increase in the expression of Kim-1 and lipocalin-2 can be seen both from the PCR and immunochemistry; furthermore, the ROC curves show that they are more sensitive and specific of gentamicin-induced kidney damage. On the contrary, serum creatinine and γ-glutamyl transferase were found to be less suitable as markers of kidney damage. The main metabolic alterations responsible for distinguishing control animals from those treated are represented by a decrease in citrate, hippurate, trigonellin and 3-indoxyl sulfate (3IS) and an increase in lactate, glucose and *N*,*N* dimethylglycin. However, we must consider that some of these metabolites (citrate, trigonellin and 3IS) are significantly altered only in high-dosage treatments and therefore cannot be considered biomarkers sensitive to, and useful in, gentamicin toxicity [16].

In Budonock’s work, a combination of gas chromatography mass spectrometry (GC/MS) and liquid chromatography mass spectrometry was used on rats between four and six weeks of age to monitor the biochemical changes induced following daily administration of nephrotoxins such as gentamicin, tobramycin and cisplatin. Many of the markers identified were associated with nephrotoxicity for the first time in the literature: polyammide, 1,5-AG, monoethanolamine, phosphates, glycylproline, glucosamine, sorbitol and 5Me THF. The analysis also confirmed some conventional markers of nephrotoxicity such as the amino acids, glucose and the osmolytes [17].

Later, Zgoda-Pols *et al.* performed a study using murine models of acute kidney injury (AKI) induced by a powerful agonist of nicotinic acid receptors (NAR), SCH900424. By means of GC-MS and LC-MS techniques, among the metabolites revealed as AKI markers, 3IS stood out. The levels of this metabolite increased markedly in rat plasma and brains. This metabolite apparently derives from tryptophan following the metabolism performed by the intestinal microbiome; the relationship between parasite and host may be an important mechanism in developing this particular chemically induced toxicity. It accumulated in the blood and urine of uremic patients due to reduced or absent urinary excretion during kidney insufficiency. Moreover, 3IS is closely connected to serum albumin (97%, the *in vitro* albumin solution) and cannot be removed with conventional methods of blood dialysis. Other plasmatic markers found to be statistically significant are: *p*-cresol sulfate (PCS, deriving from bacterial fermentation of tyrosine in the large intestine) and the catabolites of tryptophan (kynurenate, kynurenin, 3-indole lactate) which may have toxicological importance but which have not been studied in depth. The levels of urea and creatinine for low doses of SCH900424 are no different from those found in controls at any point in time. Such data suggest that 3IS may be a potential marker of toxicity both for the kidneys and the central nervous system during chemically-induced AKI [18].

In Kikuchi’s experimental study the metabolomic approach was applied in research on uremic toxins as possible markers of the effects of AST-120 (carbon particles known for their capacity to accumulate in CKD patients). The serum metabolites in healthy rats and those with CKD before and after the administration of AST-120 were analyzed by means of liquid chromatography/electrospray ionization associated with mass spectrometry (LC/ESI-MS/MS) and with analysis of the main components. Indoxyl sulfate was the main serum metabolite which made it possible to distinguish the CRF rats from the healthy ones; it was followed by hippuric acid, phenyl sulfate, 4-ethylphenyl sulfate and *p*-cresol sulfate [19].

### 3.2. Clinical Studies

A study of metabolomics applied to Traditional Chinese Medicine TCM) has been published [20]. TCM considers the kidney as the foundation of an organism’s energetic heritage since it is the base of yin and yang, the two complementary and opposite ways in which matter and energy are manifested. The pathology known as kidney-yang deficiency is considered an authentic syndrome characterized by pallor, weakness, physical and psychic asthenia, polyuria, impotence, sterility and menstrual irregularity, a sensation of cold, cold hands and feet, dyspnea, dizziness, palpitations, peripheral edemas and lumbar pain [21]. In Dong’s study of 60 subjects and others, metabolomics was used to analyze 60 patients with chronic kidney insufficiency (CKD) (30 with kidney-yang deficit and 30 without) and 30 volunteers. Metabolomics revealed a significant difference between kidney-yang patients, patients without this and healthy subjects. The key metabolites leading to this distinction are: alanine, diethyl aminomalonate, proline, citric acid, hippuric acid and histamine, arginine, proline e histidine [20].

In Gronwald’s study, NMR was used to analyze 54 samples of the urine of patients with autosomal dominant polycystic kidney disease (ADPKD) and a slight reduction in the rate of glomerular filtration. Within this cohort, 35 were receiving therapy for high blood pressure and 19 others were not. The result was compared to the NMR profiles of 46 healthy volunteers, 10 patients with ADPKD in dialysis with residual kidney function, 16 patients with kidney transplantations and 52 patients with type 2 diabetes with chronic kidney disease. Based on the NMR spectra, the authors succeeded in distinguishing patients with moderately advanced ADPKD from those with terminal ADPKD and patients with chronic kidney disease from other etiologies and healthy subjects with an accuracy above 80%. Of the 35 patients with ADPKD who were receiving treatment for high blood pressure, most showed increased excretion of proteins and also methanol. On the contrary, an increase in methanol at the urinary level was found in no control or in other groups of patients. Thanks to this study, we can conclude by stating that the NMR metabolic fingerprints of urine differentiate ADPKD from the other kidney diseases and from subjects with normal kidney function. The diagnostic and prognostic potential of these profiles calls for further evaluation [22].

The study by Nevedomskay *et al.* investigated the possibility of using the metabolomic profile to monitor the state of health of patients affected by urinary tract infections in the context of complicated and febrile forms with *E. coli.* as the main pathogen, using as study groups not only healthy subjects and affected patients, but also patients who had recovered. Thus, using the H-NMR urine profiles it was possible to classify the samples in agreement with their diagnoses and this indicates a high predictive ability. Comparing the groups of molecules derived from the different analyses, the conclusion was that some compounds (e.g., trimethylamine and acetate) are associated with the presence of the bacterial infection in the urine while others (e.g., *p*-aminohippuric acid (PAH), scylloinositol) may be considered markers of morbidity [23].

In a study on interstitial cystitis performed by Fukuy *et al.*, a new urinary biomarker, phenylacetylglutamine (PAGN), was identified for its diagnosis. The analysis was performed with the UPLC-MS method based on the metabolomic approach. The following urine samples were analyzed: 10 interstitial cystitis (IC), 10 bacterial cystitis, 10 healthy volunteers. The urinary levels of PAGN compared to creatinine (Cr) were significantly high in patients with IC (about 0.47 mg/mg Cr) and with healthy volunteers (about 0.11 mg/mg Cr). These results established the PAGN/Cr ratio at the urinary level as a new urinary marker of IC and contribute to its diagnosis [24].

A metabolomic study was carried out by Gao *et al.* in an attempt to arrive at a better understanding of the pathogenetic profile of membranous nephropathy (MN).

The diagnosis and treatment of this pathology is today based of clinical manifestations, the levels of excretion of the urinary proteins and kidney biopsies. MN patients have been clinically divided into two groups: one with low levels of urinary proteins < 3.5 g/24 h (Low Urinary Protein MN group—LUPM) and the other of patients with high levels of urinary proteins > 3.5 g/24 h (High Urinary Protein MN group—HUMP). From the study of the serum and blood samples of such patients carried out with GC-MS and then from the analysis of the data obtained with PLS-DA, an obvious metabolic difference emerged between HUPM and LUPM patients. Supporting this difference are 26 urinary and 9 serum metabolites; most of these increased in HUPM patients, both in the serum and urine. The metabolites can be divided into five groups in accordance with their trend of change in the urine and serum. Of these different metabolites, only those in the urine of HUPM patients increased significantly: the sugar alcohols, the dicarboxylic acids, the hydroxylic acids and the phenolic acids, which probably derive from the kidney metabolism, while there are no differences between the LUPM and HUPM serum samples. These metabolites are the direct result of kidney damage. The four sugar alcohols (polyols) were found to be incredibly increased in the urine of HUPM patients while their concentration had not increased significantly in the serum samples [25].

Bairaktari *et al.* [26] performed a metabolomic analysis on the urine of a 75-year-old patient presenting symptoms of bilateral myalgia in the lower limbs and weakness following a combined treatment for dyslipidemia. In this patient an increase in urinary excretion of muscle enzymes was found, but the creatinine values were normal. By means of a spectroscopic analysis based on H-NMR, important changes were noted already in the first four days. Among these was the complete suppression of hippurate excretion, reduction of the excretion in citrate (indicative of a malfunction of the kidney tubule), an increase in excretion of dimethylamine (DMA) and trimethylamine oxide (TMAO), considered markers of papillary dysfunction [27,28]. These findings indicate the presence of a generalized tubular disorder which also involves the kidney papilla. It is known that there are no markers among conventional ones in clinical chemistry that diagnose damage to the kidney papilla. TMAO on the contrary was high in the early stage of this disorder (in the urine sample at the time of admission) and then returned to reference values after myoglobin excretion had decreased [26].

In Suhre’s work, the possibility of using metabolomics in the study of diabetes and its complications through identification of a series of new and known metabolites was evaluated. The urine of 40 diabetic patients and 60 controls was analyzed with NMR and MS. The interpretation includes perturbation of the metabolic tracts correlated with the kidney dysfunction (3IS), the lipid metabolism (glycerolphospholipids, saturated fatty acids), and interaction with the gut microflora (bile acids). The results of this study suggest that the metabolic markers have the potential to identify complications correlated with diabetes already in subclinical conditions in the overall population [29].

Through analysis of the urine of patients with interstitial cystitis, bacterial cystitis and healthy subjects, Van QN *et al.*, using H-NMR and MS methods, succeeded in distinguishing different spectral patterns associated with these three groups of patients with a success rate of approximately 84% [30].

Table 1 presents a synopsis of these studies.

**Table 1:** Main studies on metabolomics in nephrology.

Kidney disease	Model	Method	Potential biomarker identified	Reference
Gentamicin nephrotoxicity	R	^1^H-NMR GC-MS	↑Kim-1, ↑lipocalin-2, ↑clustering, ↓citrate, ↓hippurate, ↓trigonellin, ↓3 indoxyl sulfate↑lactate, ↑glucose, ↑N,N dimethylglycine	[16]
Gentamicin nephrotoxicity, from cisplatin and from tobramycin	R	GC-MS LC-MS	polyamide, 1,5- AG, monoethanolamine, phosphates, glycylproline, glucosamine, sorbitol, 5Me THF, amino acids, glucose, osmolytes	[17]
AKI, *Acute Kidney Injury*	R	GC-MS LC-MS	3-indoxyl sulfate, *p-*cresol sulfate, kynurenate, kynurenin, 3-indole-lactate	[18]
CKD, *Chronic Kidney Disease*	H	not indicated	alanine, amino diethylmalonate, proline, citric acid, hippuric acid, histamine, arginine, proline and histidine	[19]
CRF, *Chronic renal failure*	R	LC/ESI-MS/MS	indoxyl sulfate, hippuric acid, phenyl sulfate, 4-ethylphenyl sulfate, p-cresol sulfate	[21]
ADPKD, *Autosomal dominant polycystic kidney disease*	H	^1^H-NMR	proteins, methanol	[22]
UTI, *Urinary tract infection*	H	^1^H-NMR	*p*-aminohippuric acid, scylloinositol	[23]
IC, *Interstitial cystitis*	H	UPLC-MS	phenylacetylglutamine (PAGN)	[24]
MN, *Membranous nephropathy*	H	GC-MS	sugar alcohols, dicarboxylic acids, hydroxylic acids, phenolic acids, cholesterol	[25]
Rhabdomyolysis	H	^1^H-NMR	↓↓hippurate, ↓citrate, ↑dimethylamine (DMA), ↑ trimethylamine N-oxide (TMAO)	[26]
Diabetes-related renal dysfunction	H	NMR MS	3-indoxyl sulfate	[29]

**Models:** R: Rats; H: Humans.

It is important to underline that some of these findings confirm experimental studies with untargeted metabolomics on the plasma and urine from wild-type and organic acid anion transporter-1 (Oat/Slc22a6) knockout mice, which identified important metabolites. For example indoxyl sulfate derives from Phase II metabolism of enteric gut precursors and accumulate in chronic kidney disease. Similarly indoxyl sulfate and kynurenine, together with xanthurenic acid, interact strongly and directly with Oat1, suggesting a complex communication between the gut microbiome, indicating the importance of Oat1 in the handling of endogenous toxins associated with renal failure and uremia [31,32]. Again, the knockout mice manifest a profound loss of organic acid transporter (e.g., *p*-aminohippurate) [33]. 

## 4. Metabolomics in Pediatric Nephrology

Despite the widespread use of biomarkers in clinical practice, there is little valid information on data relating to those concerning pediatric cases. The biomarkers found for adults are often applied to young children without considering the fact that the pathogenesis of many diseases is different and that the oncogenesis directly impacts on the evolution and response to therapy. New and innovative approaches are necessary to find other reliable and valid biomarkers for use in advanced pediatric assistance [34]. Among these new biomarkers, we have NGAL and Kim 1 now under study for their use in the diagnosis of AKI in neonates and infants. Their application for the understanding of kidney maturation is however not simple [35]. Sophisticated techniques such as proteomics and metabolomics, are at present scantily used with infants but quite promising in understanding the processes of the disease [31]. The works dealing with metabolomics in pediatric nephrology are very few.

In the experimental study by Hanna *et al.* conducted on newborn rats, nephrotoxicity induced by gentamicin was associated with different urinary patterns of metabolites. In particular, 14 parameters were significantly altered following administration of gentamicin: glucose, galactose, *N*-acetylglucosamine, myo-inositol, butanoic acid and 3-hydroxybutyrate which increased three-fold compared to normal values, followed by citrulline and pseudo-uridin which increased, but to lower levels [36]. 

In our study (Atzori *et al.*) we investigated the possibility of nephrological diseases being associated with a well-defined metabolic pattern. To that end, using ^1^H-NMR-based metabolomic methods, we analyzed the urine of 21 children affected by nephropathies (kidney dysplasia, vesicoureteral reflux, urinary tract infections and acute kidney insufficiency) and compared it with that of 19 healthy controls. From this study emerged alterations of the amino acid spectra of purine and pyrimidine, as well as alterations of the citric acid cycle [37].

Beger *et al.* applied a metabolomic analysis to the study of AKI as a complication following cardiopulmonary surgery in the pediatric age. In this study, 40 urine samples were taken from infants who had undergone surgery to correct congenital heart defects. AKI, defined as a large increase from basal serum values of creatinine, was reported in 21 infants 48 to 72 h from heart surgery. The LC/MS urine analysis of samples taken 4 and 12 h after the procedure made it possible to identify homovanillic acid (HVA-SO_4_), a metabolite of dopamine, as a new, sensitive, predictive and early AKI biomarker following pediatric heart surgery [38]. Table 2 presents a synopsis of these studies.

**Table 2:** Studies on metabolomics in pediatric nephrology (all have been performed in urine)

Kidney disease	Model	Method	Potential biomarker identified	References
Nephrouropathies	C	^1^H-NMR	hippurate, tryptophan, phenylalanine, malate, tyrosine, hydroxybutyrate, N-acetyl glutamate, tryptophan, proline	[9]
AKI (*Acute Kidney Injury*) following heart surgery	C	LC-MS	homovanillic sulfate acid (HVA-SO_4_)	[38]
Gentamicin nephrotoxicity	R	Not reported	↑↑↑glucose, ↑↑↑galactose, ↑↑↑N-acetylglucosamine, ↑↑↑myo-inositol, ↑↑↑butanoic acid, ↑↑↑3-hydroxybutyrate, ↑citrulline, ↑pseudouridin	[37]

**Models:** C: *Children* R: *Rats*.

## 5. Actual Impact of Metabolomics in Nephrology and Rare Diseases

It is not easy to compare human studies and experimental animal studies. Moreover it is difficult to compare studies in adults and studies in children and neonates. The metabolome greatly varies with age, diet, drugs assumption, lifestyle and, in adults, with gender. The newborn, infant and child are “virgin” organisms, compared to adults [39]. Thus, in general terms, the results in adults are not applicable in children, or only limitately. Firstly, because the organisms are so different and the newborn is not a small adult. Secondly, because the pathologies are different (*i.e.*, membranous nephropathy is not present in the newborn). May be that in the future metabolomics will be able to identify early and common markers of AKI, since renal injury and failure are also metabolic disorders. In this context, monitoring some metabolites such as 3-indoxylsulfate, *p*-cresol sulfate, kynurenate, kynurenine, 3-indole lactate, could be interesting and of important practical impact. Further informations could be obtained on geography of damage. For example an increase of urinary TMAO is generally related to medullary injury; similarly an increase of dimethylamine, sorbitol and myo-inositol could be expression of papillary damage. Conversely a reduction of citrate, αketogutarate and succinate is expression of tubulus proximal cells mitochondrial dysfunction, since only proximal tubule cells are able to compensate for inhibition of their mitochondrial Krebs cycle. Moreover some metabolites indicates specific pathologies: in the adult methanol is a marker of ADPKD, fenylacetyl glutamine is a marker of interstitial cystitis, and scylloinositol and *p*-aminohippuric acid are markers of urinary tract infection (UTI). Very recently in UTIs metabolomics has been defined as a “new uroscope in town” [40]. At the moment most of the studies on metabolomics include only small number of patients. Thus a problem can arise regarding the robustness of the data, and how likely it is that putative biomarkers from small metabolomics studies can be replicated in larger materials. Again for rare conditions, such replication is difficult, so it would be very important to identify strategies on how to translate these results to clinical practice. Considering rare diseases, one example could be inborn errors of metabolism (IEMs). IEMs are inborn errors of metabolism hereditary metabolic defects, which are encountered in almost all major metabolic pathways occurring in man. While many known examples are identified, some authors recently approached the problem with a new network based analysis, potentially providing a valuable tool in studying metabolic changes involved in inherited metabolic diseases [41]. The goal could be to find specific metabolites for common and rare pathologies and to prepare easy, quick and helpful tools, like “dipsticks for urine”, able to discriminate patients from controls, patients before and after a nutritional or therapeutic intervention, the right drug for the right patient (pharma-metabolomics) [15]. The goal is actually translational medicine from top research to bedside.

## 6. Conclusions

Kidney diseases are a vast and heterogeneous group of pathologies not easy to diagnose, with diagnoses often pronounced only in an advanced stage. The science of metabolomics has been practiced in the field of nephrology, although in a very primordial way, for centuries. The urine wheel published in 1506 by Ullrich Pinder, in his book *Epiphanie Medicorum* describes the possible colors, smells and tastes of urine, and uses them to diagnose disease [42].

Today “*omics*” (genomics, transcriptomics, proteomics and metabolomics) are a picture of the complexity of biological systems: simultaneous, and often noninvasive analysis of a large amount of data (the so-called “direct intelligence of data”). This means “self-organized highly interconnected networks”, systems medicine, networks medicine.

Metabolomics may represent a technology of potentially great interest in the early diagnosis of kidney diseases. Although scarce, the data on adults and the very few ones in pediatrics are quite interesting. Further studies are needed to determine the practical clinical impact of metabolomics in kidney pathologies [43,44,45,46,47,48,49,50]. In the near future, metabolomics will probably occupy a role of increasing importance, together with genomics, transcriptomics and proteomics, in the experimental and clinical study of nephrourologic diseases [51,52,53,54,55] and renal complications of metabolic diseases, such as diabetes [56].

The “multiplatform” “omic” study of urine can contribute to improving early diagnosis and the outcome of kidney diseases [57]. As a title in the recent literature suggests: “From “omics” to personalized medicine in nephrology, integration is the key”: this is even more true in pediatric nephrology. [58] In the next future each nephrologist shoud be “extremely comfortable and competent in the metabolomic space” [41].
